# Segmentation and Multimodal Characterization of Metal Particles in the Human Hippocampus Using Discrete Segmentation Algorithms and Correlation Spectral Analysis

**DOI:** 10.3390/molecules31010009

**Published:** 2025-12-19

**Authors:** Ján Pánik, Mária Ždímalová, Daniel Kosnáč, Martin Kopáni, Silvia Dulanská, Nazarii Kretsul, Michal Trnka

**Affiliations:** 1 Institute of Medical Physics and Biophysics, Faculty of Medicine, Comenius University, Sasinkova 2, 813 72 Bratislava, Slovakia; jan.panik@fmed.uniba.sk (J.P.); daniel.kosnac@fmed.uniba.sk (D.K.); martin.kopani@fmed.uniba.sk (M.K.); silvia.dulanska@fmed.uniba.sk (S.D.); 2 Department of Mathematics and Descriptive Geometry, Faculty of Civil Engineering, Slovak Technical University, Radlinského 11, 811 05 Bratislava, Slovakia; mzdimalova@gmail.com (M.Ž.); kretsul.nazar@gmail.com (N.K.); 3 Department of Radiological Technology, Faculty of Nursing and Medical Professional Studies, Slovak Medical University, Limbova 12, 833 03 Bratislava, Slovakia

**Keywords:** biomedical imaging, human hippocampus, maximum flow, metallic particles, spectral analysis

## Abstract

The accumulation of metallic micro- and nanoparticles in the human hippocampus is increasingly linked to neurotoxic processes and neurodegenerative disorders. Precise segmentation and detailed characterization of these particles are crucial to understanding their role. This study presents a novel method that combines discrete segmentation based on graph-cut theory with Dinic’s algorithm for the computation of maximum flow. The images are modeled as directed weighted graphs, with pixel intensities and gradients defining edge capacities, enabling robust segmentation in electron microscopy data. To ensure robustness, the method is validated against ground-truth masks, achieving a Dice coefficient of 0.97898 ± 0.0172 and an Intersection over Union (IoU) of 0.9609 ± 0.0326. Morphometric parameters—area, perimeter, circularity, and Feret diameters—are automatically extracted. Concurrently, elemental analysis using Energy-Dispersive X-ray Spectroscopy (EDS) reveals a heterogeneous composition, including iron-rich particles and compounds containing nickel and chromium. The observed variability highlights the importance of single-particle analysis in better understanding the neurobiological impact of metallic deposits.

## 1. Introduction

The human brain is a complex organ whose function can be influenced by the presence of various endogenous and exogenous substances. Among these are metals, which can accumulate in brain tissue in the form of nanoparticles or larger aggregates. Research suggests that certain metals, such as Fe, Ni, and Cr, play an ambivalent role in the organism: they are essential for many biological processes in trace amounts, yet their excessive presence or dysregulation of homeostasis can lead to toxic effects. Their overaccumulation, especially in key and vulnerable brain regions such as the hippocampus—a structure essential for memory and learning [1,2]—is increasingly associated with pathophysiological mechanisms that include oxidative stress, chronic inflammatory responses, mitochondrial dysfunction, and protein aggregation. These processes are considered essential in the etiopathogenesis of neurodegenerative diseases such as Alzheimer’s or Parkinson’s disease [3,4,5,6,7]. For example, nickel is known for its ability to cross the blood–brain barrier and induce neurotoxic effects by disrupting redox balance and producing excess reactive oxygen species (ROS) [8]. Iron, although essential for cellular functions, can contribute to the formation of free radicals and oxidative damage when homeostasis is dysregulated [9].

It is assumed that not only the mere presence of these metals but also their physicochemical properties in the nanoparticle form, such as size, shape, surface reactivity and degree of aggregation, can significantly modulate their interaction with neural cells, their ability to penetrate biological barriers, and their overall neurotoxic potential [10]. Understanding these relationships is essential to elucidating the mechanisms by which metallic nanoparticles contribute to neuropathology. To study these phenomena, it is imperative to develop methods that allow the precise detection, segmentation, and quantitative characterization of such metallic particles directly within biological samples. Image segmentation—the process of dividing an image into homogeneous regions based on defined criteria (e.g., intensity, texture, shape)—is the first critical step. In biomedical image analysis, graph-based methods have proven exceptionally effective [11] due to their ability to integrate both region-based information and boundary-based information. Recent applications have demonstrated their utility in complex segmentation tasks, as seen in the work of Singh et al. and others [12]. These methods transform the image into a graph where pixels represent vertices and the relationships between them are represented by edges with specific weights (capacities). The segmentation problem is then formulated as finding an optimal cut in this graph, often as the task of finding the minimum s-t cut that separates the object from the background. This approach is based on the fundamental max-flow/min-cut theorem and enables efficient determination of a global optimum for the given objective function (mathematically modeled as functional energy) [13,14,15]. The global optimum is mathematically desirable because it minimizes the total cost defined by the data (regional) and smoothness (boundary) terms, ensuring the most accurate segmentation according to the model parameters, rather than getting stuck in local minima common in other segmentation approaches. Although Dinic’s algorithm has been applied to general image segmentation, this study specifically adapts the method to the challenges of Scanning Electron Microscopy (SEM) data from hippocampal tissue, where metallic clusters range from nanometers to micrometers and often exhibit low contrast against complex biological backgrounds.

The primary aim of this study was to develop and implement a bespoke software tool utilizing a segmentation algorithm based on Dinic’s method for the analysis of imaging data from the human hippocampus containing metallic particles. In addition, we perform a detailed morphometric analysis of the segmented particles and correlate their morphological characteristics with their elemental composition obtained from concurrently measured spectra, with particular emphasis on particles containing Fe, Ni, and Cr.

## 2. Materials and Methods

### 2.1. Image and Spectral Data

In this methodology study, we analyzed a representative set of hippocampal tissue samples that contain metallic clusters. The data analyzed originate from biological and medical samples from the human hippocampus, where small clusters of metallic particles are present. These are imaging data acquired by high-resolution electron microscopy, accompanied by energy-dispersive X-ray spectroscopy (EDS/EDX) data for elemental composition analysis.

The hippocampal tissues were obtained by autopsy from the Department of Pathological Anatomy of the Faculty of Medicine, Comenius University. The samples were fixed in 10% neutral buffered formalin for 24 h and subsequently embedded in paraffin. While histological processing can potentially alter tissue ultrastructure, the metallic particles themselves are generally robust to standard fixation and embedding procedures. Paraffin blocks were sectioned using a Zeiss™ Hyrax M40 microtome (Carl Zeiss, Oberkochen, Germany). To verify section thickness and quality, samples were examined under a Nikon Eclipse 80i microscope (Nikon, Tokyo, Japan) at 20× magnification. Sections were then analyzed in a ZEISS EVO LS 15 scanning (Carl Zeiss, Jena, Germany) electron microscope at an accelerating voltage of 20 kV. EDX spectra were acquired with an AMETEK (EDAX, Pleasanton, CA, USA) silicon drift detector over the energy range 0.16–9.8 keV, with an integration time of 200 s. Standard SEM imaging conditions were maintained to ensure consistency, with pixel sizes calibrated to the magnification level used for each acquisition. In this work, we primarily analyzed data in which the presence of Fe and Ni was expected.

### 2.2. Image Segmentation Through the Graph-Cut Method

The Graph-Cut Method formulates image segmentation as a minimum cut problem in a graph [16]. It has proven to be highly effective for binary segmentation (object vs. background) in various types of image data, including medical images [14].

#### 2.2.1. Construction of the Graph for Image Segmentation

**Figure 1:**
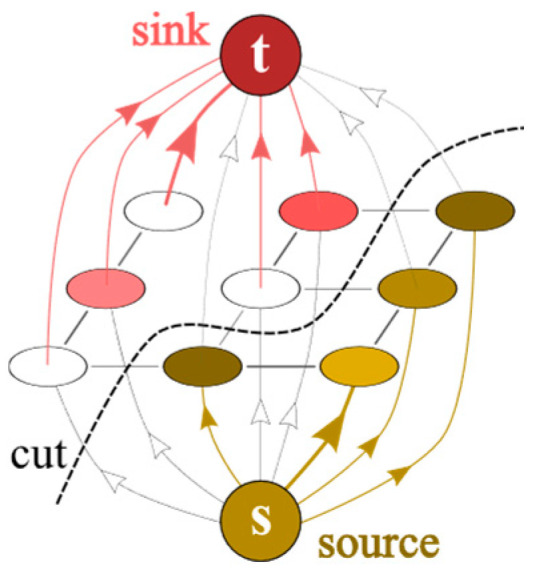
Structure of the example graph.

The core of this segmentation method is the transformation of a 2D grayscale image into a directed, weighted graph *G* = (*V*, *E*). Each pixel *p*_*ij*_ (where *i* ∈ {1, …, *m*} and *j* ∈ {1, …, *n*} for an image of size *m* × *n*) is interpreted as a vertex in the graph. The set of all pixel vertices is denoted by *P*. In addition to these vertices, two special terminal vertices are added to the graph: a source *s* (representing the “object” *O*) and a sink *t* (representing the “background” *B*). The total set of vertices is *V* = *P* ∪ {*s*, *t*} (Figure 1).

**Figure 2:**
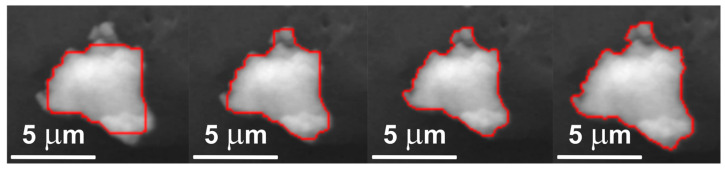
Effect of the parameter λ on image segmentation. From left to right, images are shown for λ = 0.3, 0.5, 1.0, and 100. As λ increases, segmentation accuracy visibly improves until an optimal point.

Edges *E* in the graph are of two types:

1. t-links (terminal edges): Each pixel vertex *p* ∈ *P* is connected to the source *s* by an edge (*s*, *p*) and to the sink *t* by an edge *c*(*p*, *t*). The capacities of these edges, *c*(*s*, *p*) and *c*(*p*, *t*), reflect the likelihood that the given pixel belongs to the object or background. These capacities are typically defined based on regional properties of the pixel, for example, its intensity *I*_*p*_ relative to the expected intensities of the object (*I_object_*) and background (*I_background_*). In our approach, for pixels not explicitly marked as seeds for *O* or *B*, the capacities are determined using regional cost functions *Rs*(*p*) and *Rt*(*p*) [16]:

(1) $$c\left(s,p\right)=\lambda \cdot R_{s}\left(p\right)=\lambda \cdot (M-\left|I_{object}-I_{p}\right|)$$

(2) $$c\left(p,t\right)=\lambda \cdot R_{t}\left(p\right)=\lambda \cdot (M-\left|I_{background}-I_{p}\right|)$$

where *I_object_* and *I_background_* are the mean intensities of the object and background seeds, *M* is the maximum possible intensity value (e.g., 255 for an 8-bit image) to ensure nonnegativity of the costs, and λ > 0 is a weighting coefficient that determines the relative importance of regional (t-link) information versus boundary (n-link) information. Information in this context refers to the specific contribution of pixel intensity data (regional term) versus the contribution of spatial continuity (boundary term) to the calculation of the total cost functional. The choice of λ is critical, as it balances the data term (how well the pixel fits the object/background model) and the regularization term (how smooth the object boundary should be). The influence of λ on segmentation accuracy is illustrated in Figure 2: as λ increases, segmentation accuracy improves up to an optimal value. For pixels belonging to explicitly defined object seeds *O* (e.g., user-selected), we set *c*(*s*, *p*) = *K_max_* and *c*(*p*, *t*) = 0, where *K_max_* is a sufficiently large constant (larger than the sum of all possible edge weights) to enforce the hard constraint that these pixels must belong to the object. Analogously, for background seeds *B*: *c*(*s*, *p*) = 0 and *c*(*p*, *t*) = *K_max_*. This replaces the theoretical concept of infinity used in the previous literature.

2. n-links (neighborhood edges): These edges connect neighboring pixels (e.g., using 4-neighborhood or 8-neighborhood), and their capacities reflect the dissimilarity (or cutting cost) between those pixels. The more similar two neighboring pixels are (i.e., smaller intensity difference), the higher the capacity of the n-link between them. This penalizes cuts crossing that edge and thus promotes smoothness of the object boundary (Table 1). If *p* and *q* are neighboring pixels, the n-link capacity (*p*, *q*) is defined as:

(3) $$N\left(p,q\right)=B\left(p,q\right)=exp\left(-\frac{{\left(I_{p}-I_{q}\right)}^{2}}{2\sigma^{2}}\right)\cdot \frac{1}{dist\left(p,q\right)}$$

A higher value of *N*(*p*, *q*) (i.e., a smaller intensity difference) implies that it is less likely that the edge will be cut. These edges thus encourage boundary smoothness of the segmented object. For undirected edges between neighbors *p* and *q*, one may define two directed edges (*p*, *q*) and (*q*, *p*) with the same capacity *N*(*p*, *q*), or use a single edge that allows negative flow [16].

#### 2.2.2. Segmentation via Max-Flow/Min-Cut and Dinic’s Algorithm

The image segmentation problem is formulated as finding the minimum s-t cut in the constructed graph. A minimum s-t cut is a partition of the vertex set *V* into two disjoint subsets *S* (containing the source *s*) and *T* (containing the sink *t*, with *V* = *S* ∪ *T*), such that the sum of capacities of edges going from *S* to *T* is minimized. According to the fundamental max-flow/min-cut theorem (Ford–Fulkerson theorem), the maximum flow from source *s* to sink *t* in the network is equal to the capacity of the minimum s-t cut [17]. Pixels that belong to set *S* after finding the minimum cut form the segmented object, whereas pixels in *T* form the background (Figure 1).

To compute the maximum flow (and thus the minimum cut), this work employs Dinic’s algorithm. Dinic’s algorithm operates in phases using two search strategies:

1. Breadth-First Search (BFS): Used to construct a level graph from residual graph. BFS explores the graph layer by layer, identifying the shortest path (in terms of number of edges) from the source to the sink. The level graph contains only edges that go from level *i* to level *i* + 1 and only those vertices reachable from the source *s*.
2. Depth-First Search (DFS): Used to find a blocking flow in the level graph. DFS explores as far as possible along each branch before backtracking, pushing the maximum possible flow along identified paths.

To compute the maximum, the process repeats (constructing a new level graph and finding a blocking flow) until the sink *t* is no longer reachable from *s* in the residual graph. The worst-case time complexity of Dinic’s algorithm is *O*(*V*^2^*E*), but for certain types of graphs, such as those arising in image segmentation, it can be more efficient in practice [18]. Dinic’s algorithm belongs to the class of augmenting-path algorithms and is known for its practical efficiency in image segmentation problems [19].

### 2.3. Implementation, User Interface, and Post-Processing

Based on the principles described above, a functional software tool was developed. The segmentation algorithm was implemented in C++. For the graphical user interface (GUI), the Qt library (specifically QtWidgets for windows, buttons, etc.) was used. OpenCV was utilized for basic image operations such as loading, format conversion, morphological operations, and drawing geometric elements on the output images. The application is divided into modules: ImageSegmentation.h and ImageSegmentation.cpp (GUI and event handling) and ImageSegmentationMath.cpp (core segmentation algorithm and mathematical functions). Developing such bespoke software allows optimization of algorithms with respect to the specific requirements of processing each image dataset.

In the GUI, the user first loads an image and then interactively selects a rectangular region of interest (ROI) in which segmentation will be performed. This limits computational expense, as the graph is constructed and analyzed only for pixels within the ROI. After initiating segmentation and obtaining an initial object mask, post-processing steps are applied to improve result quality:

1. Hole Filling: Small holes within the segmented object are filled using an algorithm similar to flood-fill (function fillHolesInGraph). This step is crucial for metallic particles, which may appear hollow in electron microscopy due to surface charging or uneven contrast distributions.
2. Noise Removal: Small isolated components that do not meet a size threshold are removed as noise (function removeNoiseFromGraph). Care was taken to set this threshold low enough to preserve small but significant nanoparticles while effectively removing background artifacts. To clean the resulting mask further, morphological operations such as opening and closing may also be applied.

### 2.4. Quantitative Validation

To rigorously validate the proposed segmentation method, we performed a quantitative evaluation against Ground-Truth (GT) masks. A representative subset of 12 images containing metallic particles was selected. Ground-truth masks were manually created using the Fiji software, version 2.9.0 (ImageJ distribution, version 1.54p) via the *Image* → *Adjust* → *Threshold* function followed by *Convert to mask*, ensuring a precise binary representation of the metallic particles. The predictions from our software were binarized identically.

For each GT and segmentation pair, we calculated three standard metrics: Intersection over Union (IoU), Dice Coefficient, and Pixel Accuracy. These metrics were computed using a dedicated macro script and aggregated as mean ± standard deviation.

### 2.5. Morphometric Characterization

**Figure 3:**
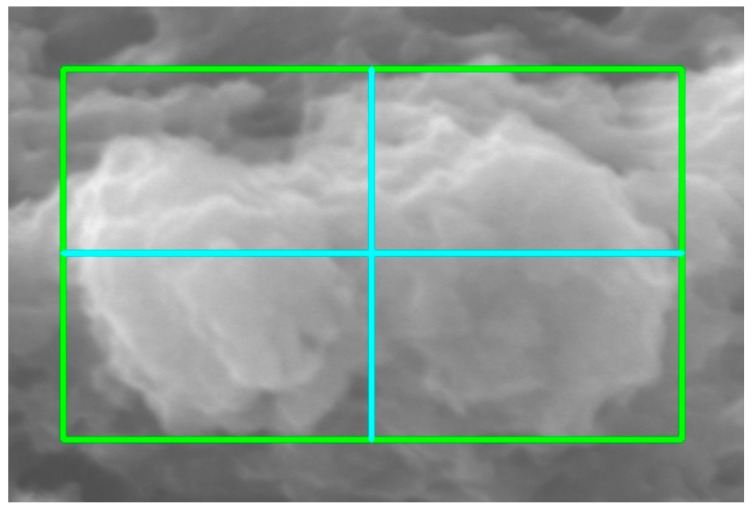
Minimum binding rectangle.

**Figure 4:**
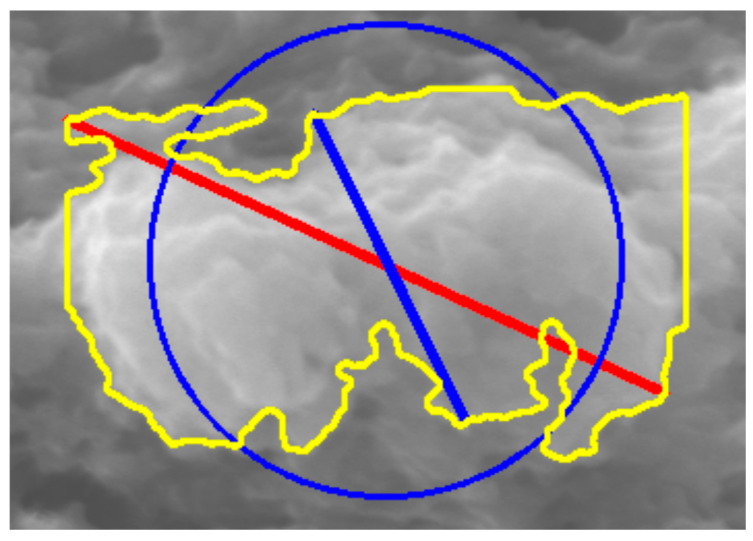
Feret diameters: longest (red) and shortest (blue).

**Figure 5:**
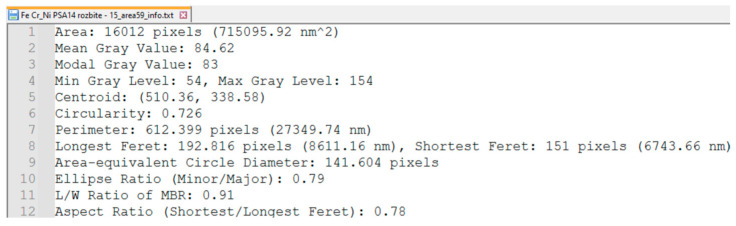
Example contents of the file INFO.TXT, listing descriptive data for an analyzed metallic particle.

For each successfully segmented particle, a set of geometric and shape parameters is calculated. Basic parameters include the area (pixels converted to real units), perimeter (contour length), centroid coordinates, mean pixel intensity, modal intensity (the most frequent pixel value), and minimum and maximum intensity values. Advanced shape parameters comprise circularity (with a value of 1 indicating a perfect circle); parameters of the fitted Legendre ellipse (major and minor axis lengths, orientation); the aspect ratio of the minimum bounding rectangle (MBR) (Figure 3); Feret diameters (the longest and shortest distances between two parallel tangents to the particle boundary) (Figure 4); and the ratio of Feret diameters. All measured parameters are stored in a text file for further analysis (Figure 5).

**Figure 6:**
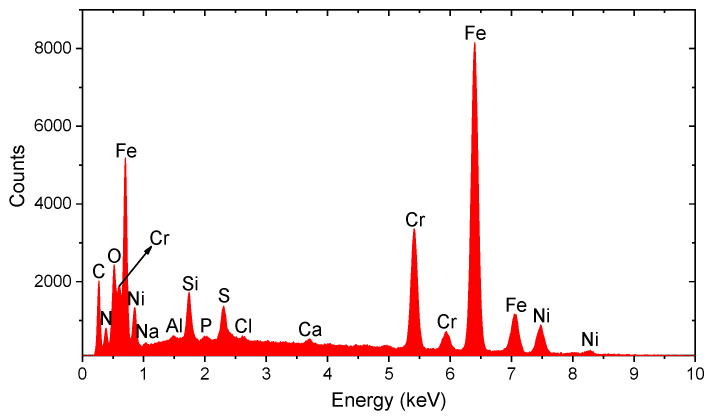
Example of each analyzed particle determined from its assigned X-ray spectrum obtained by EDX analysis.

The elemental composition of each analyzed particle is determined from its assigned EDS spectra, where the presence and relative intensity of peaks at characteristic energies correspond to specific elements (Figure 6).

### 2.6. Validation of the Segmentation Algorithm

In this work, segmentation quality was evaluated primarily through visual inspection of the resulting object masks and boundaries. For reference, standard contour detection from the OpenCV library was used to assess edge-detection quality. Comparison showed that particle boundaries segmented by the implemented Dinic algorithm closely matched the OpenCV contours, indicating high segmentation accuracy.

## 3. Results

The implemented software system was successfully applied to segment and characterize metallic particles in electron microscopy images of the human hippocampus.

### 3.1. Quantitative Validation of Results

The quantitative evaluation of the proposed segmentation algorithm demonstrated high accuracy when compared to the ground-truth masks generated in Fiji. For the analyzed image pairs (*n* = 12), the method achieved the following metrics:

- Intersection over Union (IoU): 0.9609 ± 0.0326
- Dice Coefficient: 0.9798 ± 0.0172
- Pixel Accuracy: 0.9987 ± 0.0018

These high values indicate that the graph-cut-based segmentation is robust and statistically comparable to expert-guided thresholding techniques, confirming the reliability of the method for metallic particle segmentation.

### 3.2. Morphometric Characterization of Metallic Particles

**Figure 7:**
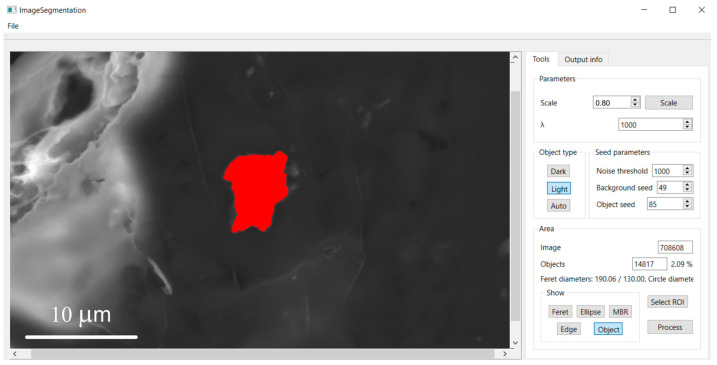
Segmented object with highlighted region indicating chemical elements after pressing the “*Object*” button.

**Figure 8:**
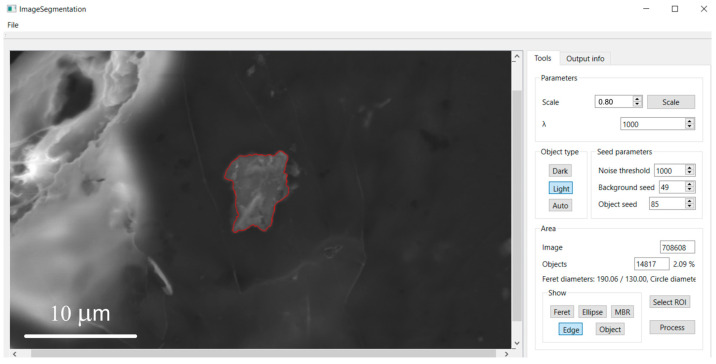
Display of the segmented object’s outlines after pressing the “*Edge*” button.

**Figure 9:**
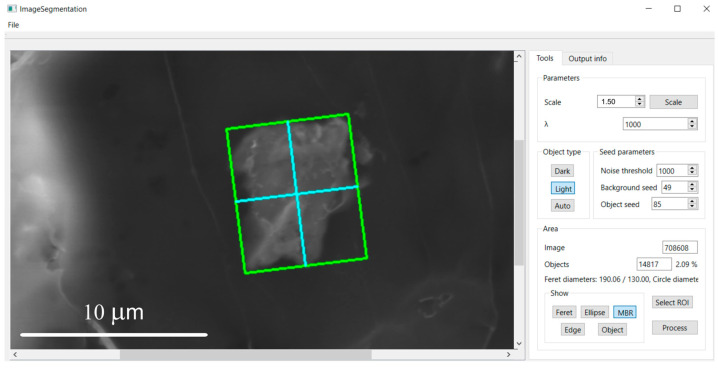
Minimum bounding rectangle of the particle shown after pressing the “*MBR*” button.

**Figure 10:**
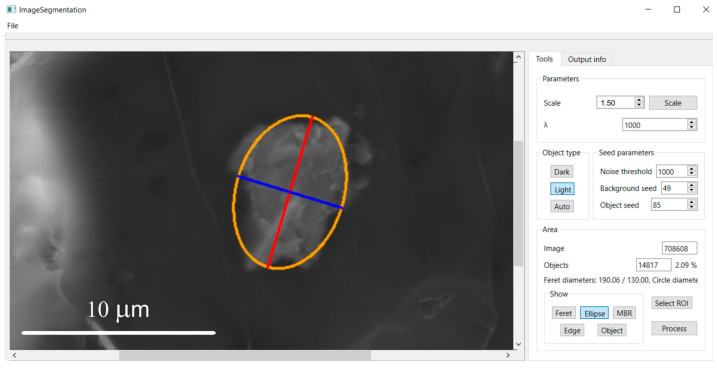
Ellipse fitted to the segmented particle after pressing the “*Ellipse*” button.

**Figure 11:**
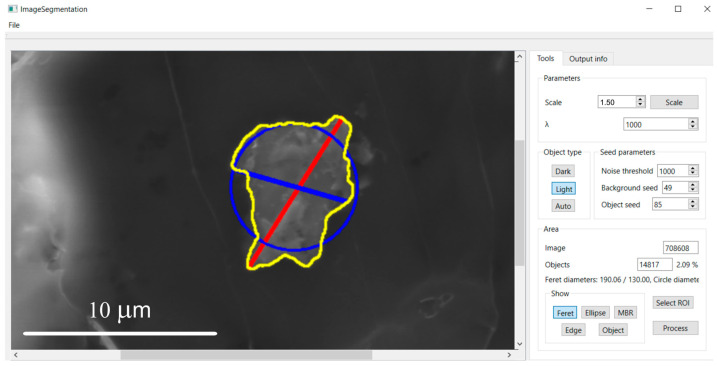
Display of the longest and shortest Feret diameter after pressing the “*Feret*” button.

The implemented software system was successfully applied to segment and characterize metallic particles in electron microscopy images of the human hippocampus. For each detected particle, the software automatically generates a set of visualizations to support detailed morphometric analysis, including:

- A colored overlay of the segmented object (Figure 7);
- A display of its contour (Figure 8);
- An overlay of the minimum bounding rectangle (Figure 9);
- A fitted Legendre ellipse (Figure 10);
- The Feret diameters (Figure 11).

**Figure 12:**
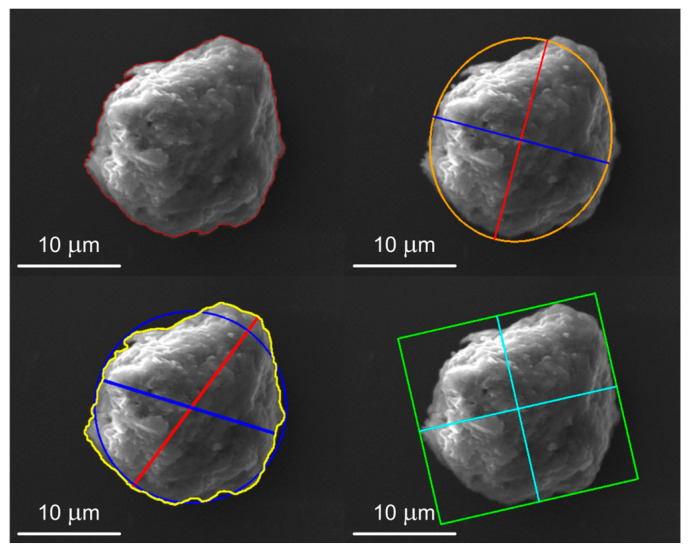
Application of segmentation algorithm to a metallic particle containing Fe and Zn.

**Figure 13:**
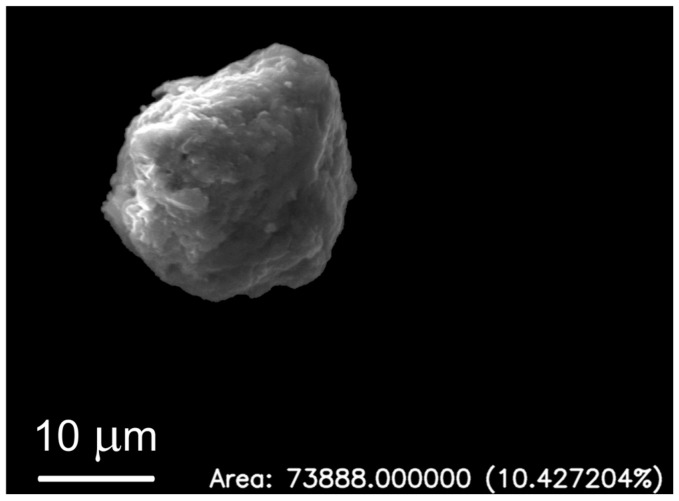
Segmented particle containing Fe and Zn without background.

Figure 12 illustrates application of the segmentation algorithm to a metallic particle containing Fe and Zn. The result is a precise delineation of the particle’s boundary, enabling its unambiguous separation from the background (Figure 13) and subsequent quantitative analysis of its shape parameters. Morphometric parameters, such as area, perimeter, circularity, and Feret diameters, are automatically saved into a structured text file (Figure 5) for further analysis.

The analyzed metallic particle, with low circularity (0.726) and moderate elongation (MBR aspect ratio = 0.91; Feret ratio = 0.78), indicates a slightly irregular shape, possibly due to surface defects or material anisotropy. Morphometric parameters for representative particles are shown in Table 2.

### 3.3. Spectral Analysis of Metallic Particles

**Figure 14:**
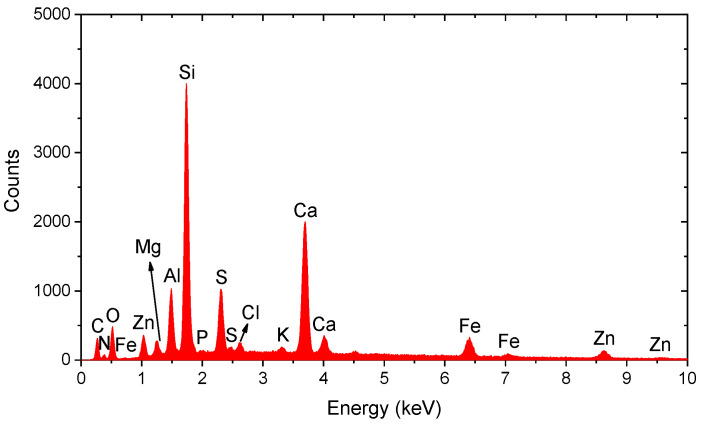
Particle containing Fe and Zn. Spectral distribution shows a dominant iron peak (~400 cps) and a secondary zinc peak (~200 cps). The Fe:Zn ratio of 2:1 indicates an iron–zinc corrosion phase.

Spectral analysis by EDS enabled detailed identification of the elemental composition of each segmented particle. For Fe-Zn particles, the spectra exhibited a dominant iron peak (~400 cps) and a secondary but noticeable zinc peak (~200 cps), corresponding to an Fe:Zn ratio of approximately 2:1, suggesting the presence of an iron–zinc corrosion phase or alloy (Figure 14).

**Figure 15:**
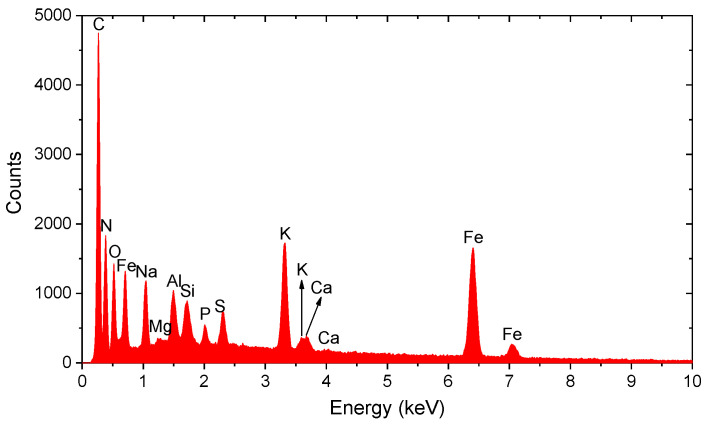
Particle containing Fe. Pronounced spectral peak (~2000 cps) confirms high content of pure iron in the analyzed particle, with no other significant metal peaks.

For Fe-dominated particles, EDS spectra exhibited high-intensity iron peak, reaching up to ~2000 cps with no significant presence of other metallic elements, indicating a high content of pure iron or iron oxides (Figure 15). Other particles with dominant Fe showed moderate signal intensity (e.g., ~1340 cps), suggesting medium-sized iron agglomerates.

The greatest variability was observed in particles containing combinations of iron, chromium, and nickel. One particle exhibited high levels of all three elements, with Fe at ~25,000 counts (6.40 keV), Cr at ~10,800 counts (5.41 keV), and Ni at ~4000–5000 counts (7.47 keV and 0.85 keV). These values indicate Fe:Cr:Ni ratios of approximately 6:2.6:1, characteristic of slag remnants or specific alloys.

Other Fe-Cr-Ni particles exhibited lower absolute intensities but similar ratios (e.g., Fe ~1350 cps; Cr ~700 cps; Ni ~200 cps), indicative of small corrosion product inclusions. Some particles displayed slight variations in elemental proportions, reflecting local heterogeneity in corrosion phases. Increased Fe:Cr:Ni ratio of approximately 4.6:2.2:1 and Fe intensity of ~2300 cps suggested a more homogeneous distribution

This variability in elemental composition and particle morphology highlights the importance of single-particle analysis for a comprehensive understanding of their characteristics in biological samples.

## 4. Discussion

In this study, we implemented and applied a methodology that combines advanced image segmentation with morphometric and spectral analysis for a detailed investigation of metallic micro- and nanoparticles in the human hippocampus. The use of Dinic’s algorithm for the maximum flow proved to be effective in accurate delineation of individual particles, even those with complex shapes or located in close proximity to surrounding tissue structures. Unlike generic segmentation tools, our implementation using Dinic’s allows for precise energy minimization specifically tuned for the intensity gradients observed in SEM images of metallic deposits. While we used OpenCV contours for a visual sanity check, the quantitative validation confirms the method’s accuracy with a Dice coefficient of ~0.98. This addresses the limitations of simple visual inspection and demonstrates the robustness of the algorithm.

### 4.1. Neurobiological Implications of Metallic Particle Presence in the Hippocampus

The detection of various metallic particles in the human hippocampus is in line with growing evidence on the role of metals in neurobiological processes and the development of neurodegenerative diseases. However, it is important to note that while these metals are associated with neurotoxicity in the literature [20,21,22,23], our findings primarily document their presence and morphology; a direct causal link to specific neurodegenerative pathways in these specific samples remains subject of future pathological correlation.

**Iron:** Particles with a dominant iron content (Figure 15) are of particular interest due to their association with oxidative stress [9,24].

**Nickel:** The detection of nickel-containing particles (Figure 6) in human hippocampal samples is especially concerning, given their proven neurotoxicity, ability to cross the blood–brain barrier and ability to induce the production of reactive oxygen species (ROS) [8,25].

**Chromium** plays a biological role in glucose metabolism. The accumulation in the hippocampus can exert neurotoxic effects and disrupt the homeostasis of other essential metals [7,26].

**Complex compounds (Fe-Cr-Ni):** The detection of particles composed of complex Fe-Cr-Ni compounds suggests an external origin, such as implant wear or environmental exposure [27,28,29,30].

### 4.2. Importance of Morphometric Analysis

Morphometric analysis, including parameters such as area, circularity, and Feret diameters (Figure 12), provides essential quantitative data on the particles. These characteristics are critical, as the physicochemical properties of micro- and nanoparticles, such as their size and shape, can significantly influence their interaction with neural cells, their ability to traverse biological barriers, and their overall neurotoxic potential [31,32]. The diversity of shapes and sizes observed in this study, ranging from nearly circular to highly elongated forms, warrants further investigation in the context of their biological reactivity and pathological consequences. Future studies could correlate these morphological features with the degree of tissue damage or the progression of neurodegenerative disease.

### 4.3. Study Limitations and Future Analysis

The primary limitation of this study is the relatively small number of samples/particles analyzed, which limits the generalizability of the biological findings. Additionally, while segmentation was validated against Fiji-based ground truth, future work should include inter-observer variability studies. Furthermore, although we used noise removal and hole filling, extremely small particles (<10 nm) might still be challenging to distinguish from background noise without further increasing resolution.

Despite these limitations, the methodology and analytical capabilities for individual particles represent a valuable contribution to understanding the complex interactions between metallic micro- and nanoparticles and human brain tissue.

## 5. Conclusions

This study implemented a novel methodology for the advanced analysis of metallic micro- and nanoparticles in the human hippocampus. Quantitative validation confirmed the high accuracy of the Dinic-based segmentation algorithm (Dice > 0.97). The developed approach enables accurate segmentation and detailed quantification of the morphometric parameters and their correlation with the elemental composition. Future studies should aim to expand the datasets and correlate morphological and spectral data with clinical findings to clarify the full extent of their impact.

## Figures and Tables

**Table 1:** Cost of Edges [16]. The Cost represents the capacity of the edge in the graph flow network.

Type	Edge		Cost
t-link	(*s*, *p*)	for *p* ∈ P/{O ∪ B}	λR_s_(*p*)
		for *p* ∈ O	*K* *_max_*
		for *p* ∈ B	0
	(*p*, *t*)	for *p* ∈ P/{O ∪ B}	λR_t_(*p*)
		for *p* ∈ O	0
		for *p* ∈ B	*K* *_max_*
n-link	(*p*, *q*)	for *p*, *q* ∈ P	*N*(*p*, *q*)

**Table 2:** Summary of morphometric parameters for representative particles.

Particle Type	Area (nm^2^)	Circularity	Aspect Ratio	Primary Elements
Fe-Zn	715,000	0.726	0.78	Fe, Zn
Fe-rich	450,000	0.85	0.92	Fe
Fe-Cr-Ni	620,000	0.65	0.6	Fe, Cr, Ni

## Data Availability

The data that support the findings of this study are available from the first author upon a reasonable request. All relevant data generated or analyzed during this study are included in this manuscript or available at Zenodo public repositories; access to additional information can be granted upon reasonable request to the authors.
